# *C. elegans* protein interaction network analysis probes RNAi validated pro-longevity effect of nhr-6, a human homolog of tumor suppressor Nr4a1

**DOI:** 10.1038/s41598-019-51649-0

**Published:** 2019-10-31

**Authors:** Bashir A. Akhoon, Shishir K. Gupta, Sudeep Tiwari, Laxmi Rathor, Aakanksha Pant, Nivedita Singh, Shailendra K. Gupta, Thomas Dandekar, Rakesh Pandey

**Affiliations:** 1 Microbial Technology and Nematology Department, CSIR - Central Institute of Medicinal and Aromatic Plants, Lucknow, 226015 India; 2 Department of Bioinformatics, Biocenter, University of Würzburg, Wuerzburg, 97074 Germany; 3 Department of Bioinformatics, CSIR-Indian Institute of Toxicology Research, Lucknow, 226015 India; 4 Department of Systems Biology and Bioinformatics, University of Rostock, Rostock, 18051 Germany; 5 BioComputing Unit, EMBL Heidelberg, Heidelberg, 69117 Germany

**Keywords:** Embryonic induction, RNAi, Computer modelling

## Abstract

Protein-protein interaction (PPI) studies are gaining momentum these days due to the plethora of various high-throughput experimental methods available for detecting PPIs. Proteins create complexes and networks by functioning in harmony with other proteins and here in silico network biology hold the promise to reveal new functionality of genes as it is very difficult and laborious to carry out experimental high-throughput genetic screens in living organisms. We demonstrate this approach by computationally screening *C. elegans* conserved homologs of already reported human tumor suppressor and aging associated genes. We select by this *nhr-6*, *vab-3* and *gst-*23 as predicted longevity genes for RNAi screen. The RNAi results demonstrated the pro-longevity effect of these genes. Nuclear hormone receptor *nhr-6* RNAi inhibition resulted in a *C. elegans* phenotype of 23.46% lifespan reduction. Moreover, we show that *nhr-6* regulates oxidative stress resistance in worms and does not affect the feeding behavior of worms. These findings imply the potential of *nhr-6* as a common therapeutic target for aging and cancer ailments, stressing the power of *in silico* PPI network analysis coupled with RNAi screens to describe gene function.

## Introduction

Most cellular processes are controlled by protein-protein interactions and networks of proteins. Therefore, the inter-relationships between proteins, rather than the individual protein, eventually determine the behavior of multiple coordinated processes in a biological system. Given the availability of large scale high-throughput proteomic and genomic interaction data, the computational methods provide us an opportunity to utilize this multiomics data for prediction of new functionality of the essential genes in these biomolecular networks. Aging happens in nearly all living organisms including humans by genetic programs and biochemical accidents such as oxidation. It implies a reduction in all provided functions over time and an increased risk for disease such as cancer. There is a clear association between aging and cancer by programmed pathways as well as by biochemical accidents. Although the intricate association between these two biological processes has been described in several studies^1–3^, many genetic details and intricacies are still unknown. There are a number of reported signaling molecules, oncoproteins and tumor suppressors including IGF1, FOXO, mTOR, and others that are well-known common targets of cancer and ageing. Interestingly, few drugs such as metformin have shown promising anti-ageing and tumor-inhibition potentials^4,5^.

*Caenorhabditis elegans* (*C. elegans*) was used as a model system to investigate unexpected new links between these two entities is a powerful approach for this question as many genes that affect tumors and ageing in mammals have orthologs representatives in *C. elegans*. The worm bearing 60–80% human gene counterparts, has already contributed enormously to our understanding of both ageing and cancer^6–8^. In fact, the tumor protective nature of longevity mutations or phytomolecules that mediate longevity has been revealed in this model system^6,9^. Therefore, finding common targets that underlie longevity and tumor suppression is an important starting point for developing novel drug molecules with therapeutic potential for both ageing and cancer. RNAi inhibition can point to the function of such targets. However, diverse cellular processes may be carried out by molecular targets disrupted by the RNAi. The complex action and effect of the knockout happens by complex networks of protein interactions. Therefore, the inter-relationships between the implied proteins, rather than the individual mRNA knockout itself, determine the behavior of the various integrated processes in a biological system.

Taking these considerations into account, we studied the protein-protein interactions (PPIs) of *C. elegans* using several computational approaches to predict genes that serve dual functions, behaving as tumor suppressor genes (TSG) and also influencing the lifespan in *C. elegans*. For validation, we combined the *in silico* predictions with RNAi tests in *C. elegans*. Our results clearly indicate the influence of top predicted targets such as, *nhr-6*, *vab-3* and *gst-23*, on the lifespan of *C. elegans* as knockdown of these genes significantly modified the *C. elegans* longevity. Moreover, orthologs of these genes are already reported as tumor suppressor genes in humans however we suggest their additional involvement in complex network processes implied in aging thereby sharpening the pleiotropic effects of these genes.

## Material and Methods

### Network reconstruction

The experimentally determined physical and genetic PPIs of *C. elegans* present in the BioGRID database version 3.2.110. (https://thebiogrid.org/) were used to parse PPI of *C. elegans* genes associated with aging and tumor suppression. The BioGRID ver. 3.2.110 consist 8459 PPIs of *C. elegans*. To reconstruct the aging network, we used the *C. elegans* longevity genes reported in GenAge database (http://genomics.senescence.info/genes/)as query to mine their curated PPIs from BioGRID. Further, to create a TSG network in *C. elegans*, first we used OrthoMCL version 2.0.9 (http://orthomcl.org/orthomcl/) to identify *C. elegans* orthologs of human TSGs present in TSGene database (https://bioinfo.uth.edu/TSGene/), with stringent BlastP cutoff of 1e-5 and MCL inflation parameter of 1.5. OrthoMCL uses the reciprocal best hits strategy and then exploits similarity measures to identify clusters of orthologs and paralogs, using a Markov clustering (MCL) algorithm^10^. The prepared list of TSG in *C. elegans* was manually curated, mapped to gene symbols and the genes specifically reported as TSG in *C. elegans* were added to the list. To avoid the possibility of missing interactions during offline parsing of BioGRIDdata we used all the synonyms of genes in the TSG query list. Both the aging and TSG networks were reconstructed and merged with Cytoscape software version 2.8.1^11^.

### Network analysis

The graphical properties of the aging, TSG and merged aging-TSG PPI networks were analyzed using Network Analyzer plugin (ver. 2.7) of Cytoscape. The graph theoretical analysis was performed on each network to determine the network diameter, the mean path length, degree and centrality of nodes. The topologically important nodes for the integrity of networks were defined based on degree distribution and betweenness values. The aging and TSG network were compared to identify the *C. elegans* TSG present in aging network, which were probable candidates for affecting lifespan on *C. elegans*. The TSG that were not previously reported for involvement in aging were ranked based on the degree and betweeness rank in the merged aging-TSG network. The genes were further reranked by Rankmin score.

1 $$Ran{k}_{\min }(i)=Ran{k}_{de}(i)+Ran{k}_{be}(i)$$

where ‘i’ indicates the ith protein in the reconstructed PPI, Rankde is the ranking of protein ‘i’ based on degree score and Rankbe is the ranking of proteins ‘i’ based on betweenness score estimated by cyto-Hubba plugin of Cytoscape^12^.

### Nematode strains and RNAi knockdown

The *C. elegans* strains used in this work include wild-type Bristol N2, GR1307: daf-16 (mgDf50), DA1116: eat-2 (ad1116), EU1: skn-1 (zu67), EU31: skn-1(zu135), VC199: sir-2.1(ok434) IV, PS3551: hsf-1 (sy441), PS746: let-23(sy97) II.

RNAi analysis of vab-3, nhr-6 and gst-23 mutants was performed as described previously^13^. Briefly, RNAi clones were grown overnight in LB with 50 µg/ml ampicillin and then seeded into the NGM agar plates containing 1 mM isopropylthiogalactoside (IPTG) and 25 ug/ml carbenicillin. Plates were kept for 7 days before feeding and then L4-stage larvae were transferred to the NGM plates seeded with RNAi induced bacteria. The worms were allowed to lay eggs at 20 °C and the progeny was transferred onto another plate seeded with the same bacteria. The process was repeated till the 6th generation L4-stage larvae.

### Lifespan analysis

Age-synchronized RNAi treated L4 molts were transferred to RNAi agar plates containing 50 mM 5-fluorodeoxyuridine (FudR). Worms were kept at 20 °C and transferred to fresh agar plates, containing their respective RNAi bacteria, every 2–3 days to avoid contamination and to ensure the continued efficacy of RNAi knockdown. Worms feeding on bacteria carrying the empty vector (L4440) was used as control. The survival of worms was scored on the basis of body movement using touch-provoked method.

### Oxidative stress assay

For oxidative stress assay, worms were maintained for 6 days until progeny production ceased. Subsequently, 90 nematodes were transferred to fresh NGM plates containing 10 mM paraquat and checked daily for viability^14^.

### Quantification of ROS formation

The ROS assay was performed using around 300 young (day 1) adult worms as described^15^. The Cary Eclipse fluorescence spectrophotometer (Agilent Technologies) was used to capture the fluorescence spectral measurements at the excitation and emission wavelength of 485 nm and 535 nm, respectively.

### Real-time qPCR analysis

The worms grown at 20oC were washed thrice and RNA was isolated using RNAzol RT (Molecular research Centre, Cincinnati, Ohio). The cDNA was synthesized using cDNA synthesis kit (Invitrogen, USA) according to manual instructions. Primers for specific amplification of genes were designed using the NCBI primer designer. The mRNA expression of target genes was quantified in comparison to housekeeping gene β-actin (act-1) using SYBR green (Takara Bio SYBR Premix Ex Taq, DSS Takara Bio India Pvt. Ltd.) detection method on a fast real-time PCR system (Applied Biosystems 7900 HT). qPCR data was analysed using the ΔΔCt relative quantitation method^16^.

### Statistics

Statistical analyses of all data relevant to lifespan and stress resistance assays was carried out by log rank test using the Kaplan–Meier survival assay in MedCalc software. ASSISTAT 7.7 beta statistical assistance software was used to perform ANOVA (Analysis of Variance). The data was considered statistically significant at p value less than 0.05. The number of asterisks represents the following: * denotes p < 0.05 and ** denotes p < 0.01.

## Results and Discussion

Protein-protein interactions are essential for life. Studying such interactions primarily relies on sophisticated experimental methods such as RNAi. RNAi has been used widely to decipher unreported functions. Advances in the *in silico* network biology have made it possible to use protein-protein interaction networks for better understanding of cellular functions in both physiologic and pathologic conditions. These *in silico* omics methods can help us in making functional predictions based on the principle that a protein with unreported function may interact with previously characterized proteins and accordingly we can assign its function. These functional predictions complement approaches such as RNAi that probe gene function directly by interfering on the transcript level.

### *In silico* PPI analysis reveals involvement of *vab-3*, *gst-23* and *nhr-6* in *C. elegans* longevity

**Figure 1:**
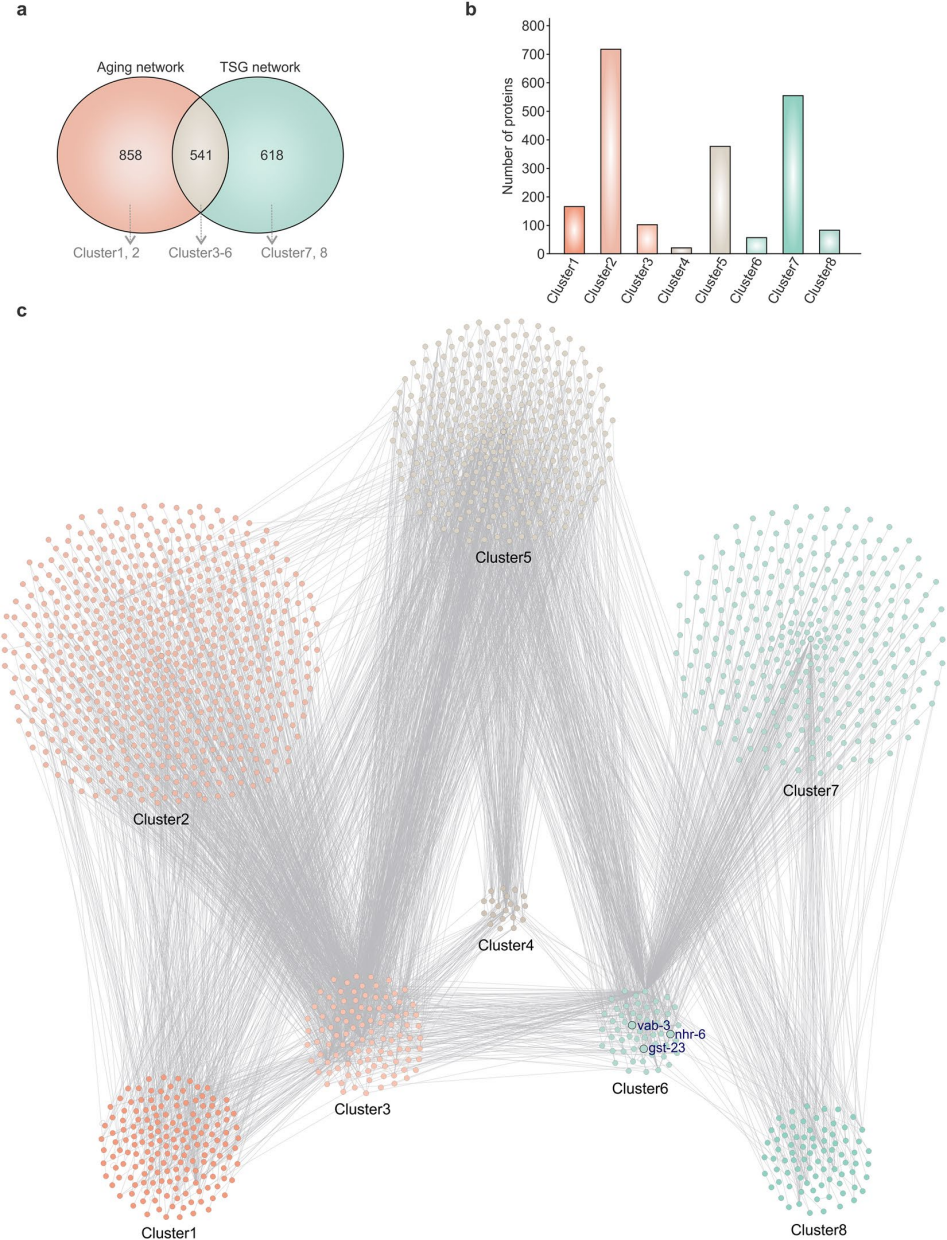
Protein-protein interaction network. (**a**) Venn diagram showing overlap between aging network and tumor supressor gene (TSG) network. Combined network is splitted into clusters. (**b**) Number of genes into each cluster. (**c**) Protein-protein interaction network of aging and human tumor supressor gene orthologs of *C. elegans*. Only the primary connectors of *nhr-6* are highlighted. The annotation of the nodes can be accessed from the XGMML (extensible graph markup and modelling language) network Supplementary File 1. Network is organized into eight clusters. Cluster1 - proteins associated with aging. Cluster2- proteins exclusively interacts with aging proteins but not with the TSG orthologs. Cluster3- connector proteins annotated to be involved in aging. Cluster4- proteins included exclusively in intersection of aging and TSG network but connectors are annotated as both aging and TSG. Cluster5- proteins included exclusively in intersection of aging and TSG network but conntectors are not annotated as aging or TSG. Cluster6- connector proteins annotated as human TSG orthologs. Cluster 7- proteins exclusively interacts with tumor supressor proteins but not with the aging associated proteins. Cluster8- proteins associated with tumor supression.

With the aforesaid considerations in mind, we utilized experimentally validated, physically interacting protein pairs obtained from the BioGRID database to analyze the mediators between TSG and aging networks in *C. elegans*. The BioGRID database (https://thebiogrid.org/) has collaborated with WormBase (http://www.wormbase.org/) for data sharing and well-curated protein-protein interaction (PPI) information. We queried 741 aging related (lifespan increasing and/or decreasing) genes of *C. elegans* from the GenAge database (http://genomics.senescence.info/genes/) and retrieved 2279 interactions associated with 1399 unique interactor proteins to reconstruct the aging relevant network. Furthermore, to determine the PPIs associated with *C. elegans* TSG (tumor suppressor gene) homologs, we first identified 464 worm orthologs of 221 human TSG in 196 ortholog clusters, out of 716 human TSG present in the TSGene database (https://bioinfo.uth.edu/TSGene/) by the robust orthology detection pipeline OrthoMCL (http://orthomcl.org/orthomcl/). The list was updated manually, enriched with literature based *C. elegans* TSG, and queried with the BioGRID database, which resulted in 1388 interactions mediated by 1159 interactors in the reconstructed TSG network. The merged aging-TSG network hosts a total of 2017 unique *C. elegans* genes mediating 3249 PPIs (Fig. 1, Supplementary File 1).

A number of cancer genes affect aging, however only few reports that TSG may also affect aging are available^17^. The topological properties of a PPI network give a comprehensive view of the network. This helps to identify the central components important for the network connectivity. Some of our identified TSG orthologs are already known to be involved in aging and had many network connections with aging genes in the aging network. Therefore, we hypothesized TSG may also impact the lifespan of *C. elegans*. Firstly, we manually organized the network into eight functional clusters (all proteins for each cluster are given in Supplementary Table 1). Second, within the aging gene network, we annotated 74 TSGs (Fig. 1c, Cluster 4 and Cluster 6) of which 25 already have a reported role in aging as curated from the literature. Next, we ranked the rest of 49 genes according to their overall importance in the merged aging-TSG network using important network parameters such as their node degree distribution and their “betweenness centrality”. Network analysis and re-ranking based on Rank_min_ score (see online Methods and materials) revealed the genes *vab-3*, *gst-23*, and *nhr-6* as the top three TSG candidates that should impact *C. elegans* aging and be tested by RNAi.

### A priori probabilities to hit a longevity gene

Our approach was systematically built up to identify by a guilt-by-association approach combined with semantic similarity filters the high scoring genes with a high functional score to really be connected to longevity or functionally similar to lifespan reduction genes (LRGs). The Supplementary File 2 gives a detailed analysis, all network raw data are given in Supplementary File 3. In the following the key results of this analysis are given so that the reader can better appreciate the power of the *in silico* approach used to identify longevity genes:

In particular, the probability of any randomly picked *C. elegans* gene to reduce lifespan upon inhibition is low (0,35%) and hence picking three random genes each having a reduction in lifespan has an *a priori* very low probability of (0.0035)^3^ = 0.000000042875 so 4.2 × 10^−8^. The probability of any randomly picked *C. elegans* gene with high network importance to reduce lifespan upon inhibition is also low, the fraction of LRGs among hub nodes is about 5% (see Supplementary File 2) and to just randomly point at hub nodes and to get in a row three LRGs is again quite low, in this case 0.00014, so 14 in 100.000. Moreover, the guilt-by-association approach shows that we actively enriched specific functions in each cluster and the clusters have highly significant different gene functions enriched (see the cross-correlation table in Supplementary File 2). Furthermore, to subclassify genes further we used a semantic similarity score according to gene ontology. Hence, the genes picked come from clusters enriched in longevity genes were further differentiated by being those genes with the highest scores to be good targets, beyond 0.9 (Supplementary File 2).

### RNAi confirm pro-longevity effects of *vab-3*, *gst-23* and *nhr-6* in *C. elegans*

We reasoned that if the three predicted genes (*vab-3*, *gst-23*, and *nhr-6)* are involved in aging, the RNAi inhibition of these genes should affect the lifespan of *C. elegans*. The transcription factor VAB-3, an ortholog of vertebrate Pax-6, is a member of the paired domain containing pax-6 gene family. Pax-6 is involved in various developmental processes, functions as a tumor suppressor in glioblastoma and also limits prostate cancer development^18,19^. Likewise, GSTP1 (a member of the glutathione S-transferase family), an ortholog of *gst-23*, is a promising cancer biomarker and its hyper-methylation was seen in multiple types of cancers such as breast, prostate, and hepatocellular carcinoma^20^. The loss of function of GSTP1 was found to increase the susceptibility to chemically-induced skin and lung cancers^21^. Similarly, Nr4a1 (Nur77), an ortholog of *nhr-6*, has been reported to regulate tumorigenesis by maintaining the homeostasis of proliferation, apoptosis, and differentiation^22^. The involvement of *nhr-6* in cell proliferation and cell differentiation during the development of the spermatheca and spermatheca–uterine valve of *C. elegans* has also been observed^23^.

**Figure 2:**
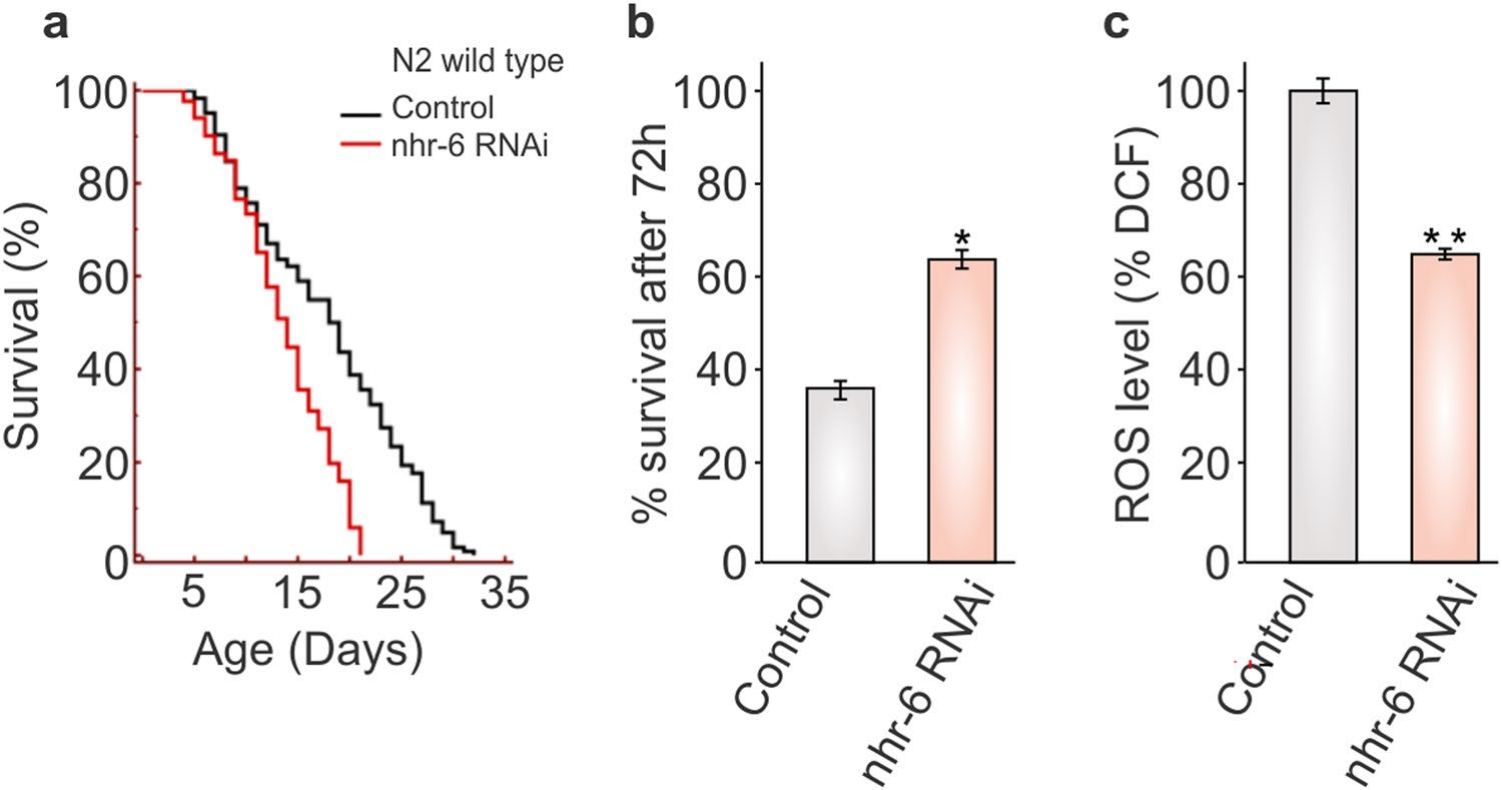
Effect of *nhr-6* RNAi on lifespan and stress level in wild type *C. elegans*. (**a**) Lifespan curves of wild type N2 worms fed with *nhr-6* RNAi. Complete lifespan data are presented in Table 1 including numbers of worms and S.E. measured. The lifespan data were statistically analyzed by log rank test using the Kaplan–Meirsurvival analysis in Medcalc 12.7.7.0 software and Graphpad prism 5. Differences between the data were considered significant at P ≤ 0.05. (**b**) Effect of *nhr-6* RNAi on paraquat induced oxidative stress level in wild type worms (n = 90). Bars represent means ± SE. **P < 0.01. (**c**) ROS levels of treated (*nhr-6* RNAi) and untreated worms. *P < 0.05.

The silencing of gene expression by RNAi has evolved as a powerful approach to delineate the gene function. Although, not all organisms are RNAi-capable but *C. elegans* has been widely used as an exemplary model organism for RNAi experiments due to its rapid take up and spread of triggering dsRNAs^24^. The short generation time and ease of culture makes it an ideal model organism for an RNAi experiment, particularly for lifespan experiments. Further, the availability of large libraries of engineered ‘RNAi foods’ has significantly increased the robustness of RNAi in *C. elegans*. Since, the ‘feeding RNAi technique’ has the potential to provide large number of desired RNAi worms required for lifespan analysis; we performed an RNAi screen of the three predicted genes (*nhr-6*, *gst-23*, and *vab-3*) in *C. elegans* by feeding *the respective* RNAi clones using the well-established RNAi protocol^13^. As shown in Fig. 2a and Table 1, the lifespan of *C. elegans* was greatly reduced by 23.46, 15.27, and 13.29%, after feeding the RNAi of *nhr-6, gst-23*, and *vab-3*, respectively. These findings validate the *in silico* predictions and show that all the three identified genes act as potential pro-longevity genes and are involved in the *C. elegans* lifespan maintenance. Since *nhr-6* showed great impact on the *C. elegans* lifespan, we were interested to further analyze the role of *nhr-6* in *C. elegans* longevity.

**Table 1:** Lifespan data: effects of *nhr-6, vab-3* and *gst-23* RNAi on *C. elegans*.

Genotype	Mean Lifespan	S.E	Number of worms	% Change	*P* value
wild type N2	**17.83**	**0.57**	**136**		
*nhr-6* (RNAi)	**13.64**	**0.25**	**130**	**23.46**	**P < 0.0001**
*vab-3* (RNAi)	**15.10**	**0.32**	**127**	**15.27**	**P < 0.0001**
*gst*-*23* (RNAi)	**15.45**	**0.47**	**126**	**13.29**	**P < 0.0001**

### *nhr-6* silencing promotes stress tolerance in *C. elegans*

*nhr-*6 belongs to the family of nuclear receptors (NRs) that are the most abundant ligand gated transcriptional regulators in metazoans, conserved across species and are known to regulate metabolism, reproduction, homeostasis, and key developmental signalling pathways. Three complete metazoan genome sequences compared revealed different numbers of predicted NR genes which include 270 for the nematode (*C. elegans*), 21 for the fruit fly (*Drosophila melanogaster*), and ~50 for the human^25^. The ability of NRs to regulate transcription is associated with and based on their susceptibility to the influence of specific small binding ligands. Some of the known NR binding ligands include thyroid, steroid, xenobiotic and metabolic intermediates^23^. Several studies have taken the advantage of the free living nematode model *C. elegans* and revealed role of few NRs in the lifespan regulation, metabolism, stem cell proliferation, development, nutrient sensing, and longevity^26,27^.

In PPINs, the topological characteristics of PPIs reflect the functionality of the interacting genes and the important genes are more likely to be well connected and globally centered in the PPIs. To get functional insights of *nhr-6*, we analyzed the network up to secondary connections by considering *nhr-6* as seed and then performed the Gene Ontology (GO) annotations. Statistically significantly over-represented GO slim terms were obtained by Fisher’s exact test followed by correcting the multiple testing errors using an FDR (false discovery rate) cutoff 0.5. The over-represented terms include MAPK cascade, signal transduction, cell communication, DNA repair, metabolic and developmental process, and response to stress. This is in line with the fact that aging is a multifactorial process which affects several biological functions. The detailed GO results of *nhr-6* primary network and *nhr-6* secondary network is listed in the Supplementary Table 2. Notably; none of the toxicity related terms were over-represented which discards the possibility of induced toxicity by gene RNAi inference.

Besides the *in silico* predictions, multiple studies have reported the major role of stress in lifespan regulation^28,29^. Therefore, we first examined whether *nhr-6* contributes to stress resistance in worms. The oxidative stress was induced in worms using reactive oxidants generator paraquat and the effect of *nhr-6* on the *C. elegans* oxidative stress resistance was analyzed by calculating the viability of worms. Next, we measured the total ROS production in *C. elegans* with the help of a dye (the dye H_2_DCF-DA). Our measurements showed that RNAi of *nhr-6* increases resistance to paraquat induced oxidative stress by 27.8% (Fig. 2b). As shown in Fig. 2c, the ROS production of worms was also decreased by 33.4%. These results suggest that *nhr-6* regulates oxidative stress resistance in worms. The findings also demonstrate that stress resistance is not intimately linked to increased lifespan and their overlap is inexact. An inverse correlation between lifespan and ROS production across a variety of species was also reported by Lambert and Brand while conducting study on mitochondria and aging^30^. In fact, researchers were unable to find any lifespan extension after supplementation of some anti-oxidants such as N-acetylcysteine and vitamin C^31,32^. These findings make it clear that increased resistance to oxidative stress is not sufficient for longevity assurance.

### *nhr-6* modulates SKN-1 and HSF-1 activity in *C. elegans*

The *skn-1*/*nrf-2* pathway and the heat shock response (*hsf-1*) are cellular defense systems which are directly influenced by the transcription factors SKN-1 and HSF-1^33,34^. In a situation of cellular stress, primarily the s*kn-1*/*nrf-2* comes to rescue, followed by *hsf-1*, which neutralizes proteotoxicity thus saving the cellular proteome. HSF-1, which encodes a heat shock factor ortholog, regulates the expression of stress induced genes required for the maintenance of protein homeostasis and development in organisms^34^.

**Figure 3:**
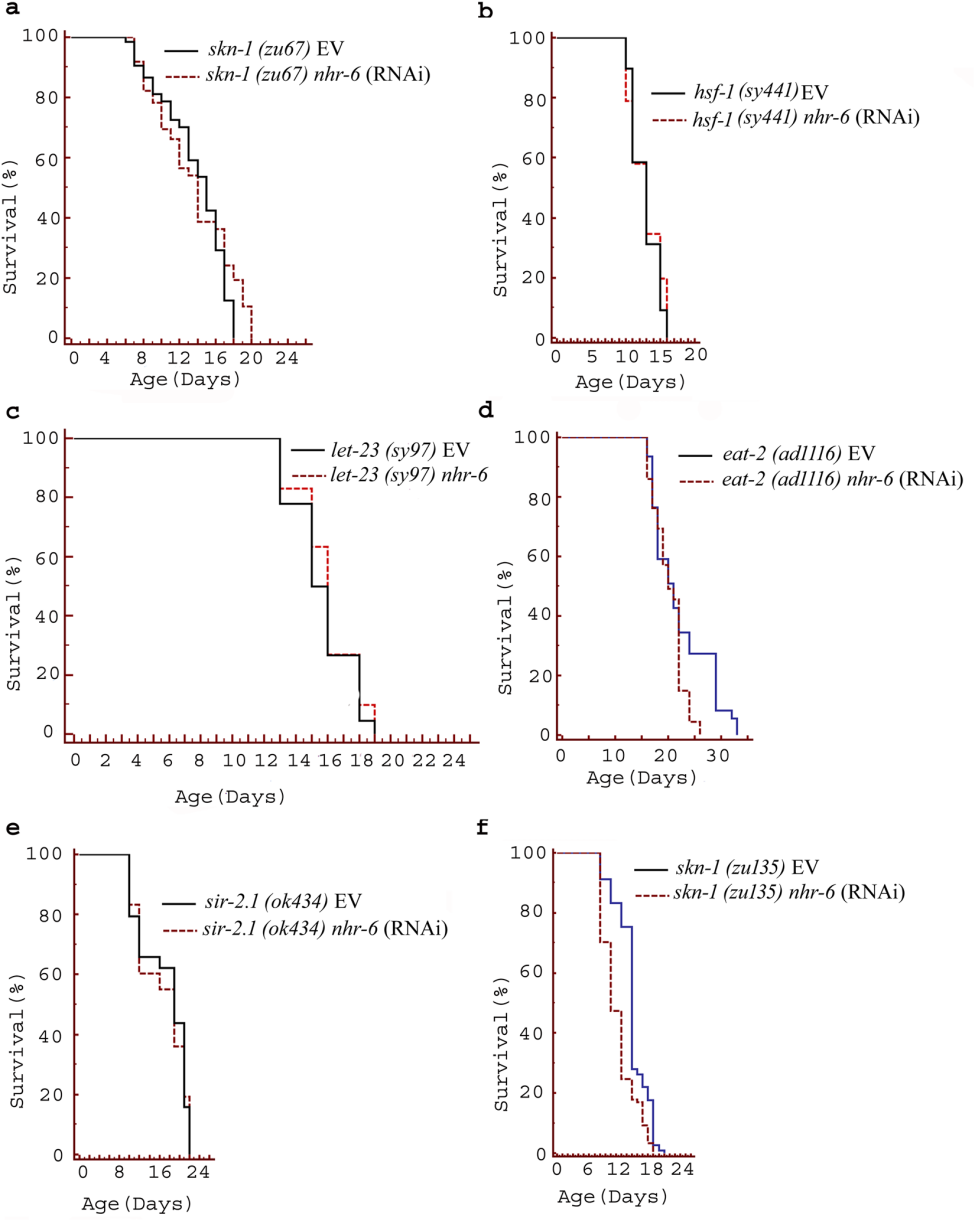
Lifespan analysis on RNAi knockdown of *nhr-6* in different mutant strains of *C. elegans* relevant to lifespan and stress resistance (detailed data including numbers of worms and S.E. are given in Table 2). The lifespan data were statistically analyzed by log rank test using the Kaplan–Meir survival analysis in Medcalc 12.7.7.0 software. Differences between the data were considered significant at P ≤ 0.05.

The gene silencing by feeding RNAi has been widely used by the researchers to silence a gene of interest in a mutant strain (Minois *et al*.^35^; Sutphin *et al*.^36^). In this study, *nhr-6* RNAi feeding was given to *skn-1* (*zu67*) and *hsf-1 (sy441)* mutant animals to trigger the RNAi knock-down of the candidate *nhr-6* gene in these mutants. The mean lifespan of the *nhr-6* RNAi fed animals (*skn-1* and *hsf-1)* was then compared with the control worms to elucidate the effect of *nhr-6* knock down on *skn-1* and *hsf-1*. The *nhr-6* failed to augment the mean lifespan in *skn-1* (*zu67*) and *hsf-1 (sy441)* mutants which suggests interaction of *nhr-6* with the stress response pathways regulated by *skn-1* and *hsf-1* (Fig. 3a,b; Table 2).

**Table 2:** Lifespan analysis of *C. elegans* mutant strains fed with *nhr-6* RNAi.

Genotype	Mean Lifespan	S.E	Number of worms	% Change	*P* value
*skn-1* (*zu67*) EV	**13.748**	**0.319**	**127**		
*skn-1(zu67)nhr-6* (RNAi)	**13.661**	**0.385**	**124**	**0.29**	**0.0425**
*skn-1(zu135)* EV	**11.322**	**0.283**	**118**		
*skn-1 (zu135)nhr-6* (RNAi)	**13.974**	**0.272**	**114**	**23.39223**	**<0.0001**
*sir-2.1(ok434)* EV	**17.122**	**0.526**	**82**		
*sir-2.1(ok434)nhr-6* (RNAi)	**16.641**	**0.532**	**78**	**2.8**	**0.8094**
*hsf-1* (*sy441*) EV	**12.779**	**0.223**	**77**		
*hsf-1* (*sy441*) *nhr-6* (RNAi)	**12.84**	**0.25**	**81**	**0.23**	**0.4368**
*let-23(sy97)* EV	**15.633**	**0.195**	**90**		
*let-23(sy97) nhr-6* (RNAi)	**15.927**	**0.283**	**41**	**1.83**	**0.3162**
*eat-2* (*ad1116*) EV	**20.21**	**0.157**	**114**		
*eat-2* (*ad1116*) *nhr-6* (RNAi)	**22.17**	**0.229**	**110**	**9.54**	**0.0006**

**Figure 4:**
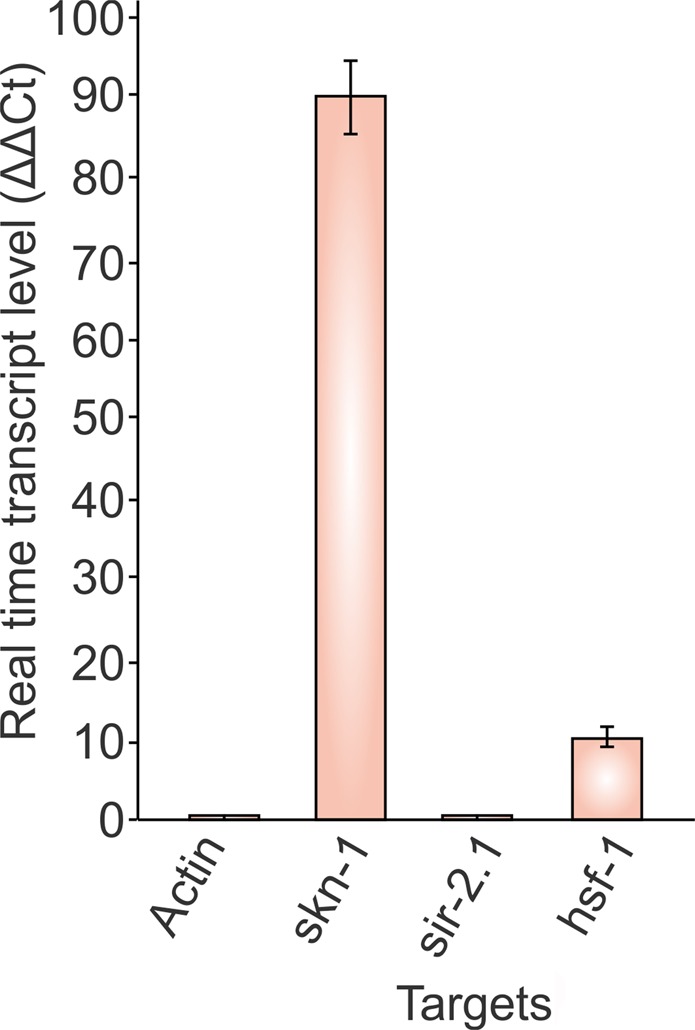
Real time quantification of mRNA expression of gerontogenes on RNAi knockdown of *nhr-6*. The *nhr-6* knockdown in N2 wild-type *C. elegans* up regulated the expression of *skn-1* and *hsf-1* in comparison to control worms fed on empty vector. However, the expression of *sir-2.1* was seen unaffected. The *β-actin* gene served as endogenous control.

qRT-PCR is one of the most common tools used to measure gene expression changes in living organisms. We used this technique to determine the gene expression of *skn-1* and *hsf-1* in *nhr-6* RNAi treated worms. It was observed that *nhr-6* knock down up-regulated 90-fold and 11-fold the expression of *skn-1* and *hsf-1* in N2 wild-type worms in comparison to empty vector fed worms (Fig. 4), thus validating the role of *nhr-6* in regulating expressions of *skn-1* and *hsf-1* that are key regulators of stress response in *C. elegans*. We hypothesize that in the absentia of *nhr-6*, *skn-1* and *hsf-1* comes into action to maintain normal homeostasis and development in worms. We assume that the increased expression of these genes might be one of the probable reasons for the increased oxidative stress resistance seen in *nhr-6* RNAi treated worms.

### *nhr-6* acts in a pathway with *let-23* to regulate lifespan in *C. elegans*

Since MAPK cascade was recognized as one of the over-represented terms in GO annotations, we evaluated the possible interaction of *nhr-6* with MAPK by analyzing the impact of *nhr-6* RNAi on the lifespan of *let-23* gene mutant *let-23(sy97)* EV. *let-23* receptor/mpk-1 MAP kinase signalling pathway is responsible for vulva development in *C. elegans*^9^. The *nhr-6* knock down failed to prolong the lifespan of *let-23* gene mutant (Fig. 3c, Table 2), validating the *in silico* predictions and suggesting that *nhr-6* may influence lifespan, at least in part, by interacting with *let-23*.

### *nhr-6* extends *C. elegans* lifespan independently of dietary restriction

Dietary restriction (DR) is one of the major phenomenons that regulate aging in a variety of species including *C. elegans*^37^. *C. elegans eat-2* encodes a ligand gate ion channel subunit similar to nicotinic acetylcholine receptors (nAChR), which functions in the pharyngeal muscle and regulate the pharyngeal pumping rate^38^. The *eat-2* gene plays a key role in the regulation of normal lifespan, feeding behavior, and defecation^39^. *eat-2* mutants have defective pharyngeal pumping that limits their food intake and therefore these animals are considered a genetic *model* of DR. We asked if *eat-2* is necessary for *nhr-6* mediated longevity in *C. elegans* and examined the lifespan of *eat-2 (ad1116)* mutants supplemented with *nhr-6* RNAi. *nhr-6* knock down showed an increment of 9.54% in the mean lifespan of *eat-2* mutants in comparison to worms fed on an empty vector (control), suggesting that *eat-2* does not participate in the *nhr-6* mediated longevity in *C. elegans* (Fig. 3d, Table 2). In addition, several studies have reported *sir-2.1* (*C. elegans* homolog of *Sir2*) to function similar to *eat-2* and its overexpression has been shown to account for *C. elegans* dietary restriction-induced longevity^40–42^. We therefore examined the effect of *nhr-6* RNAi on the lifespan of *sir-2.1(ok434)* mutant and found that *nhr-6* knock down also augmented the mean lifespan in *sir-2.1* mutant worms (Fig. 3e, Table 2). As the increment in the *sir-2.1* mutant lifespan was marginal, we tested the *sir-2.1* gene expression in wild-type worms treated with *nhr-6* RNAi. *nhr-6* RNAi knock down worms failed to demonstrate any significant up-regulation or down-regulation of *sir-2.1* expression, indicating that *sir-2.1* is not necessary for *nhr-6* mediated longevity in *C. elegans*. The cap ‘n’ collar transcription factor SKN-1 is known to encode a bZip transcription factor orthologous to the mammalian Nrf (Nuclear factor-erythroid-related factor) transcription factors, which are known to regulate development in organisms (Tullet *et al*., 2008). SKN-1 located in the ASI neurons sense food availability and has been shown necessary for lifespan extension in response to a variety of DR methods^33^. To test this hypothesis, we used a loss of function mutant strain of *skn-1, skn-1*(*zu135*). Interestingly, we find that *nhr-6* RNAi significantly extended the lifespan of this mutant strain (Fig. 3f, Table 2), indicating that *skn-1* is not required for *nhr-6* mediated longevity in *C. elegans*. These observations signify that *nhr-6* did not affect the feeding behavior of worms to mediate lifespan.

Altogether, our results confirm the role of some tumor suppressor genes such as *nhr-6, vab-3 and gst-23* in aging and demonstrate the potential of omics methods, particularly PPI network analysis, to uncover the new functionality of human candidate genes. The conserved molecular architecture of these genes raises the prospective to understand the molecular mechanism that governs the expression of these genes and to regulate them by some exogenous drug or plant-based molecules for human health benefits as these genes are already reported as tumor suppressors in humans.

## Supplementary information

Supplementary Information File 1. The merged aging-TSG network. It hosts a total of 2017 unique C. elegans genes mediating 3249 PPIs.

Supplementary Information Table 1. The network is organized into eight functional clusters of proteins. All proteins for each cluster are given.

Supplementary Information File 2. A priori probabilities to hit a longevity gene (detailed analysis).

Supplementary Information File 3. Network raw data for the probability estimates (for the analysis given in Supplementary File 2).

Supplementary Information Table 2. GO Results for the nhr-6 network. Detailed GO results of nhr-6 primary network and nhr-6 secondary network are given. 

## Data Availability

All data analysed in this paper are made fully available and are contained in the manuscript and its supplementary files.
