# Supplementary material for: C. elegans protein interaction network analysis probes RNAi validated pro-longevity effect of nhr-6, a human homolog of tumor suppressor Nr4a1
Source: Sci Rep. 2019 Oct 31;9:15711. doi: 10.1038/s41598-019-51649-0 (PMC6823380; doi:10.1038/s41598-019-51649-0)
Supplement: Supplementary file 3 — Supplementary Information File 2. A priori probabilities to hit a longevity gene (detailed analysis). [file 41598_2019_51649_MOESM3_ESM.pdf]

Supplementary Table 2: GO-Slim annotation (biological process) of primary and secondary network of tumor suppressor *nhr-6*

C. elegans protein interaction network analysis probes RNAi validated pro-longevity effect of nhr-6, a human homolog of tumor suppressor Nr4a1

Bashir A. Akhoon[1†], Shishir K Gupta[2†], Sudeep Tiwari[1], Laxmi Rathor[1], Aakanksha Pant[1], Nivedita Singh[3], Shailendra K Gupta[4], Thomas Dandekar[2,5,*], Rakesh Pandey[1,*]

[1] Microbial Technology and Nematology Department, CSIR - Central Institute of Medicinal and Aromatic Plants, Lucknow 226015, India

[2] Department of Bioinformatics, Biocenter, University of Würzburg, Wuerzburg, 97074, Germany

[3] Department of Bioinformatics, CSIR-Indian Institute of Toxicology Research, Lucknow, 226015, India

[4] Department of Systems Biology and Bioinformatics, University of Rostock, Rostock, 18051, Germany

[5] BioComputing Unit, EMBL Heidelberg, Heidelberg, 69117, Germany

[*] Corresponding authors: Rakesh Pandey: Tel: +91 9453023391; Fax: +91 0522 2716141; Email: r.pandey@cimap.res.in and Thomas Dandekar: Tel:+49 931 31 84551; Fax: +49 931 31 84552; Email: dandekar@biozentrum.uni-wuerzburg.de

GO-Slim annotation (biological process) of primary and secondary network of tumor suppressor *nhr-6*

Primary Network

PANTHER GO-Slim Biological Process	#	#	expected	Fold Enrichment	+/-	raw P value	FDR
MAPK cascade	107	5	.19	26.78	+	1.40E-06	3.13E-04

intracellular signal transduction	394	6	.69	8.73	+	5.97E-05	2.67E-03
signal transduction	849	7	1.48	4.72	+	5.88E-04	2.19E-02
cell communication	957	7	1.67	4.19	+	1.18E-03	3.31E-02
cellular process	4274	20	7.46	2.68	+	4.20E-06	3.14E-04
DNA repair	102	3	.18	16.85	+	8.04E-04	2.57E-02
metabolic process	3682	17	6.43	2.65	+	4.70E-05	2.63E-03
developmental process	465	5	.81	6.16	+	1.25E-03	3.10E-02
Unclassified	13969	11	24.38	.45	-	3.67E-06	4.11E-04

Secondary Network

**PANTHER GO-Slim Biological Process**	**#**	**#**	**expected**	**Fold Enrichment**	**+/-**	**raw P value**	**FDR**
cell-matrix adhesion	9	4	.31	12.75	+	7.15E-04	3.91E-03
negative regulation of apoptotic process	44	10	1.53	6.52	+	1.10E-05	1.23E-04

regulation of biological process	1134	74	39.52	1.87	+	6.07E-07	9.71E-06
biological regulation	1410	81	49.14	1.65	+	1.90E-05	2.03E-04
apoptotic process	104	12	3.62	3.31	+	5.44E-04	3.05E-03
cell death	106	13	3.69	3.52	+	1.89E-04	1.24E-03
death	106	13	3.69	3.52	+	1.89E-04	1.28E-03
developmental process	465	48	16.21	2.96	+	1.83E-10	8.18E-09
transmembrane receptor protein tyrosine kinase signaling pathway	24	5	.84	5.98	+	2.59E-03	1.18E-02
cell surface receptor signaling pathway	272	21	9.48	2.22	+	1.46E-03	7.28E-03
signal transduction	849	67	29.59	2.26	+	2.71E-09	1.01E-07
cell communication	957	71	33.35	2.13	+	8.22E-09	2.63E-07
cellular process	4274	272	148.95	1.83	+	3.98E-25	4.46E-23
chromatin assembly	24	5	.84	5.98	+	2.59E-03	1.16E-02
organelle organization	608	41	21.19	1.93	+	1.37E-04	1.06E-03
cellular component organization	898	66	31.30	2.11	+	4.21E-08	9.42E-07
cellular component organization or biogenesis	1007	72	35.09	2.05	+	2.91E-08	7.23E-07
RNA localization	48	9	1.67	5.38	+	1.12E-04	9.29E-04
localization	1148	66	40.01	1.65	+	1.44E-04	1.04E-03
MAPK cascade	107	18	3.73	4.83	+	2.06E-07	3.85E-06
intracellular signal transduction	394	33	13.73	2.40	+	9.52E-06	1.18E-04
cell-cell adhesion	30	5	1.05	4.78	+	6.00E-03	2.40E-02

cell differentiation	135	20	4.70	4.25	+	2.77E-07	4.78E-06
cellular component morphogenesis	112	15	3.90	3.84	+	2.48E-05	2.52E-04
anatomical structure morphogenesis	31	6	1.08	5.55	+	1.36E-03	6.93E-03
phospholipid metabolic process	119	15	4.15	3.62	+	4.68E-05	4.19E-04
lipid metabolic process	324	25	11.29	2.21	+	4.26E-04	2.45E-03
primary metabolic process	2940	176	102.46	1.72	+	1.01E-12	5.67E-11
metabolic process	3682	234	128.32	1.82	+	9.97E-21	7.45E-19
phosphate-containing compound metabolic process	1011	71	35.23	2.02	+	8.08E-08	1.65E-06
system development	64	8	2.23	3.59	+	2.85E-03	1.25E-02
nucleobase-containing compound transport	82	10	2.86	3.50	+	1.06E-03	5.50E-03
transport	1035	60	36.07	1.66	+	1.98E-04	1.27E-03
nuclear transport	67	8	2.33	3.43	+	3.68E-03	1.56E-02
fatty acid metabolic process	114	13	3.97	3.27	+	3.60E-04	2.12E-03
chromatin organization	145	16	5.05	3.17	+	1.12E-04	9.64E-04
cellular component movement	158	16	5.51	2.91	+	2.75E-04	1.66E-03
DNA repair	102	10	3.55	2.81	+	4.56E-03	1.86E-02
DNA metabolic process	213	20	7.42	2.69	+	1.29E-04	1.03E-03
nucleobase-containing compound metabolic process	1510	98	52.62	1.86	+	8.67E-09	2.43E-07
RNA splicing, via transesterification reactions	107	10	3.73	2.68	+	6.20E-03	2.43E-02
RNA metabolic process	840	56	29.27	1.91	+	1.01E-05	1.19E-04

regulation of phosphate metabolic process	215	20	7.49	2.67	+	1.45E-04	1.02E-03
regulation of catalytic activity	157	14	5.47	2.56	+	1.99E-03	9.69E-03
regulation of molecular function	175	15	6.10	2.46	+	2.00E-03	9.54E-03
mRNA splicing, via spliceosome	119	10	4.15	2.41	+	1.20E-02	4.54E-02
cell cycle	397	32	13.84	2.31	+	3.10E-05	3.02E-04
regulation of transcription from RNA polymerase II promoter	234	16	8.16	1.96	+	1.27E-02	4.74E-02
transcription from RNA polymerase II promoter	356	26	12.41	2.10	+	7.77E-04	4.15E-03
transcription, DNA-dependent	455	35	15.86	2.21	+	3.36E-05	3.14E-04
cellular protein modification process	545	37	18.99	1.95	+	2.46E-04	1.53E-03
protein metabolic process	1042	61	36.31	1.68	+	1.43E-04	1.07E-03
cellular component biogenesis	423	28	14.74	1.90	+	2.08E-03	9.69E-03
biosynthetic process	1028	67	35.83	1.87	+	2.53E-06	3.34E-05
response to stress	420	27	14.64	1.84	+	3.29E-03	1.42E-02
response to stimulus	1465	87	51.06	1.70	+	2.40E-06	3.36E-05
intracellular protein transport	383	24	13.35	1.80	+	7.78E-03	3.00E-02
protein transport	399	26	13.91	1.87	+	3.80E-03	1.58E-02
nitrogen compound metabolic process	1413	87	49.24	1.77	+	7.30E-07	1.09E-05
Unclassified	13969	342	486.83	.70	-	4.43E-29	9.92E-27
