# Supplementary material for: C. elegans protein interaction network analysis probes RNAi validated pro-longevity effect of nhr-6, a human homolog of tumor suppressor Nr4a1
Source: Sci Rep. 2019 Oct 31;9:15711. doi: 10.1038/s41598-019-51649-0 (PMC6823380; doi:10.1038/s41598-019-51649-0)
Supplement: Supplementary file 4 — Supplementary Information File 3. Network raw data for the probability estimates (for the analysis given in Supplementary File 2). [file 41598_2019_51649_MOESM4_ESM.pdf]

Supplementary File 2: Network evaluation and statistical tests for the manuscript

C. elegans protein interaction network analysis probes RNAi validated pro-longevity effect of nhr-6, a human homolog of tumor suppressor Nr4a1

Bashir A. Akhoon[1†], Shishir K Gupta[2†], Sudeep Tiwari[1], Laxmi Rathor[1], Aakanksha Pant[1], Nivedita Singh[3], Shailendra K Gupta[4], Thomas Dandekar[2,5,*], Rakesh Pandey[1,*]

[1] Microbial Technology and Nematology Department, CSIR - Central Institute of Medicinal and Aromatic Plants, Lucknow 226015, India
[2] Department of Bioinformatics, Biocenter, University of Würzburg, Wuerzburg, 97074, Germany
[3] Department of Bioinformatics, CSIR-Indian Institute of Toxicology Research, Lucknow, 226015, India
[4] Department of Systems Biology and Bioinformatics, University of Rostock, Rostock, 18051, Germany
[5] BioComputing Unit, EMBL Heidelberg, Heidelberg, 69117, Germany

[*] Corresponding authors: Rakesh Pandey: Tel: +91 9453023391; Fax: +91 0522 2716141; Email: r.pandey@cimap.res.in and Thomas Dandekar: Tel:+49 931 31 84551; Fax: +49 931 31 84552; Email: dandekar@biozentrum.uni-wuerzburg.de

Network evaluation and statistical tests

The network (Figure 1) we reconstructed consists clusters of aging (both lifespan increasing and decreasing clustered together) and putative tumor-suppressor genes (TSGs) coded proteins. We focused on the proteins that had TSGs annotation but might also be involved in aging based on their connectivity with the aging annotated proteins. We further tested the priori probabilities to hit a longevity associated gene coding protein.

Probability of any randomly picked *C. elegans* gene to reduce lifespan upon inhibition

We used GCF_000002985.6_WBcel235 assembly of *C. elegans* that contains 30180 proteins. In GenAge database, a total of 105 proteins/genes were annotated as lifespan reduction genes (LRGs). Therefore, the probability of picking up a lifespan reduction gene (LRGs) in 30180 genome proteins will be 0.35%. Since there are LRGs in the genome, the odds of picking up any other random gene will be 99.65%. Our network consists a total of 2017 proteins. Taking the network as background the probability of picking up an LRGs will be 5.21%. Since there were 105 LRGs in the network the odds of picking up any other random gene will be 94.79%.

Probability of any randomly picked *C. elegans* gene with high network importance to reduce lifespan upon inhibition

The topological analysis of the network revealed a total of 290 high network importance proteins. Among these 91 were solely classified as hub if they connect with 5 or >5 other proteins, 51 were solely appeared within top 10% bottlenecks, and 148 were within top 10% bottleneck but also had 5 or >5 connectors. We further identified with in the set of annotated LRGs total 44 proteins had high network importance (32 were solely hub, 1 solely appeared within top 10% bottleneck, and 11 were within top 10% bottleneck but also classified as hub. Notably 61 LRGs had no network importance. Therefore, the probability of picking up high network importance annotated LRGs will be 41.91%. Since there were 44 LRGs in the network that also had network importance, the odds of picking up any other random gene with high network importance to reduce lifespan will be 58.1%.

This analysis showed that a good proportion of LRGs had high network importance. In our network out of 290 high network importance proteins, 44 were already annotated as LRGs coding proteins in GenAge database. Therefore, within rest of 246 proteins we can expect around 103 proteins (41.91% of 246) might have function

in lifespan reduction. On randomly picking up the 1727 proteins (reducing the 290 high network importance proteins from the total network proteins) with no network importance, the probability of picking up LRGs will be only 3.53%. Conclusively, picking the high network importance gene highly increased the chances of success during the experiments.

Although these were a random expectation and following points should be considered before any biological interpretation about a priori probabilities

- The network is limited to aging genes and TSGs, although each gene in the network may have more binding partners (outside our network). This can subsequently impact the classification of network importance genes.

- New candidate gene for lifespan reduction might have or not have direct interactions with the known LRGs. This might be due to indirect regulation or unknown PPIs.

Prospects of randomly picked *C. elegans* gene from cluster 8, 1, 4 or 6 to reduce lifespan upon inhibition

To calculate these, we build a new cluster (cluster 9: derived from cluster 1, 3 and 4) in the network consisting the experimentally verified lifespan reduction genes coded proteins as annotated in GenAge database (Fig. S1, Supplementary File 3). According to the Guilt by Association (GBA) theory, gene/proteins function can be predicted from the networks based on the function of their connectors (Gillis and Pavlidis, 2012). We first annotated the number of proteins in each cluster connecting with LRGs (cluster 9), then applied Kruskal-Wallis test and Pairwise Wilcox tests to analyse the similarities between clusters based on their connections with LRGs. The result showed the clusters were significantly different from each other based on their connection with LRGs in most of the cases (p-value < 0.05) (see matrix below).

	Cluster1	Cluster2	Cluster3	Cluster4	Cluster5	Cluster6	Cluster7
Cluster2	1.50E-06	-	-	-	-	-	-
Cluster3	0.00015	0.43006	-	-	-	-	-
Cluster4	0.03011	0.69131	0.97994	-	-	-	-
Cluster5	3.30E-11	9.20E-06	0.43006	0.74129	-	-	-
Cluster6	1.90E-07	0.01101	0.37032	0.66358	0.73241	-	-
Cluster7	<2e-16	<2e-16	<2e-16	<2e-16	<2e-16	<2e-16	-
Cluster8	3.60E-05	3.90E-14	2.00E-11	8.10E-10	<2e-16	3.80E-15	-

Accordingly, it can be expected that the proteins drawn from different clusters might have different aging related function if they connect with LRGs. Additionally, as many proteins within the same cluster may have several different functions (multifunctional proteins; can be annotated by gene ontology (GO) annotations) so their potential to alter lifespan can also be different. Indeed, it also cannot be ignored that many connections in the network might be missing therefore applying GBA to predict the lifespan reduction potential may be oversighted. To overcome this, we measured the semantic similarity between the individual proteins of all the clusters and LRGs (cluster 9). We annotated the GO of all the proteins from different clusters and further

identified the GO categories overrepresented for cluster 9 and then accessed the semantic similarities score (for molecular function) using recently published GOGO algorithm (Zhao and Wang, 2018). It should be noted that many proteins could not be annotated with GO class and many proteins could not be scored because of no similarity of GO terms between the individual protein and cluster 9. Although the annotated proteins are enough to access the lifespan reduction possibilities. Among the top three genes that we have discussed in the manuscript, only nhr-6 was accessed by this as gst-23 could not be annotated by GO-term and vab-3 lacks semantic similarity with LGRs.

We identified the semantic similarity of nhr-6 GO terms with LRGs as **0.9**. This indicated the higher probability of lifespan reduction on removing this protein. Here we compare this score with the proteins from the other clusters

Cluster 8 consist of protein annotated as TGSs coded proteins. The maximum score we noticed was 0.42 (Fig. S2) which indicates their functional difference with LRGs.

Cluster 1 consist of proteins annotated to be involved in aging. The maximum score we noticed was 0.9 for xbp-1 (Fig. S3). Xbp-1 is annotated as aging gene in the GenAge database but its influence on longevity is still unannotated (http://genomics.senescence.info/genes/search.php?search=xbp-1). Based on the scoring, we believe that this gene might also be involved in lifespan reduction. Supporting this, it has been reported that the overexpression of xbp-1 in muscle cells reduces the lifespan by 25% (https://www.ncbi.nlm.nih.gov/pubmed/23791175). Beside this, other proteines in the cluster scored < 0.6.

We could only score cep-1 in cluster 4 which showed 0.38 semantic similarity score with LRGs. Comparatively with nhr-6 the score was very less to claim any lifespan reduction involvement of cep-1.

In cluster 6 proteins, we found sex-1 with the 0.9 score like nhr-6 which is also the member of same cluster (Fig. S4). Among other proteins, let-418 was scored 0.81. However, let-418 mutants showed the increased lifespan attributed to its regulatory functions (De Vaux et al., 2013). For the other proteins in this cluster the score was < 0.4. Evidently, plx-1, one of the bottom proteins in the cluster 6 based on its topological importance, was scored only 0.08.

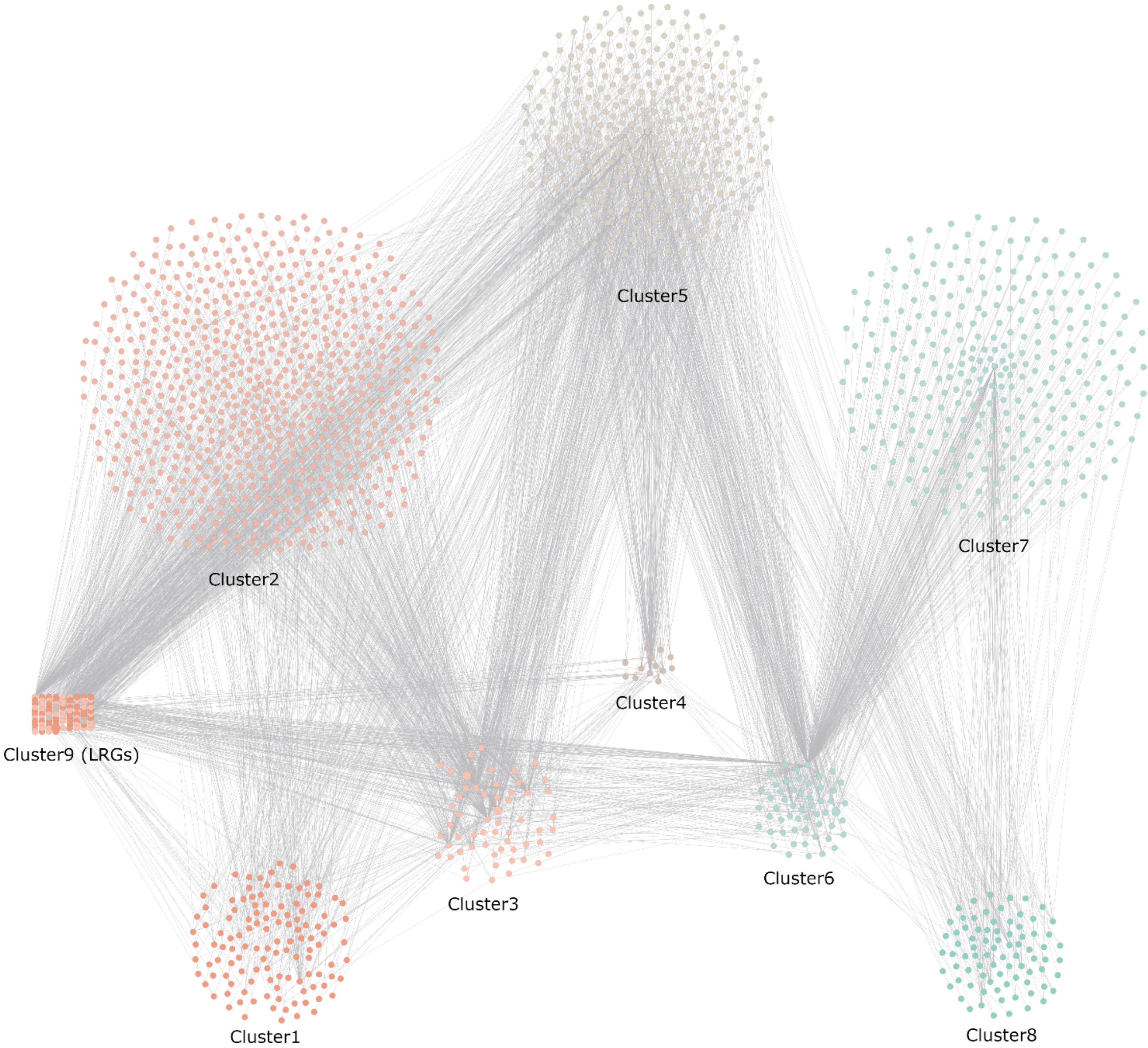

Figure S1. Protein-protein interaction network of aging and human tumor supressor gene orthologs of *C. elegans*. Network is organized into nine clusters. Eight clusters are same as shown in Fig. 1c. Cluster 9 is added which is derived from Cluster 1, 3 and 4 and contains lifespan reduction genes (LRGs). The annotation of the nodes can be accessed from the XGMML (extensible graph markup and modelling language) network Supplementary File 3.

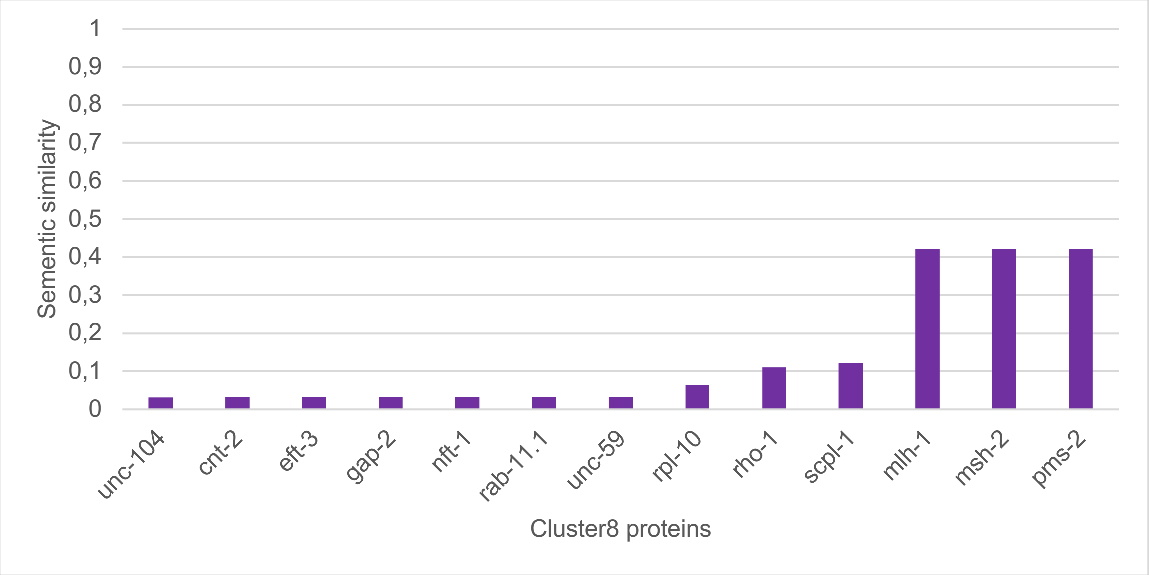

Figure S2. GO semantic similarity between cluster 8 proteins and LRGs.

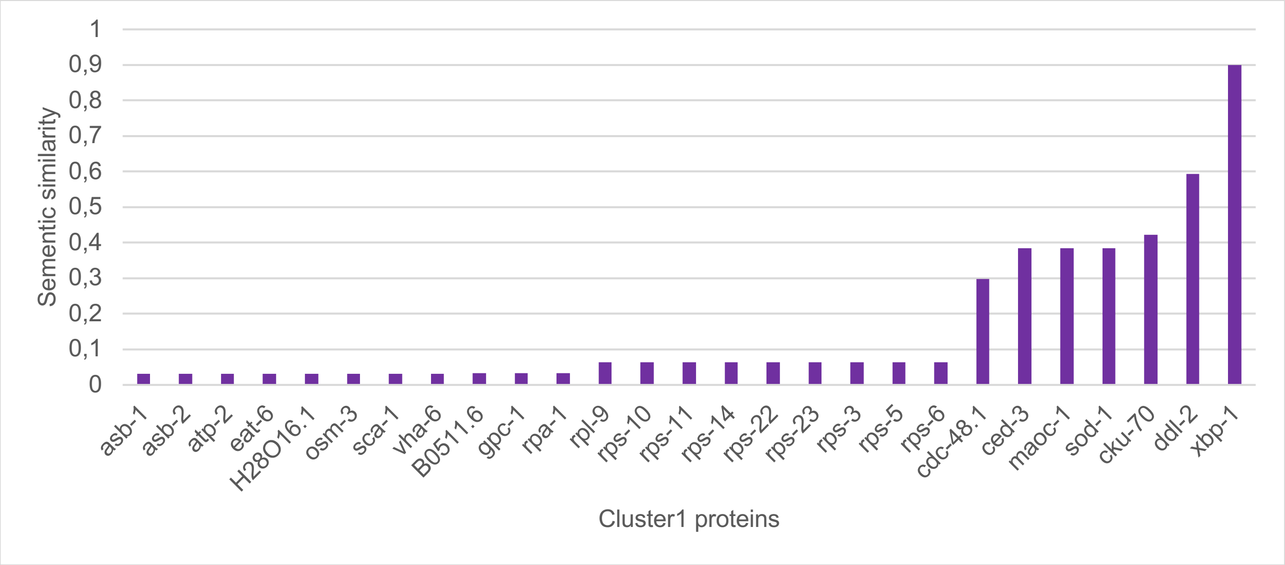

Figure S3. GO semantic similarity between cluster 1 proteins and LRGs.

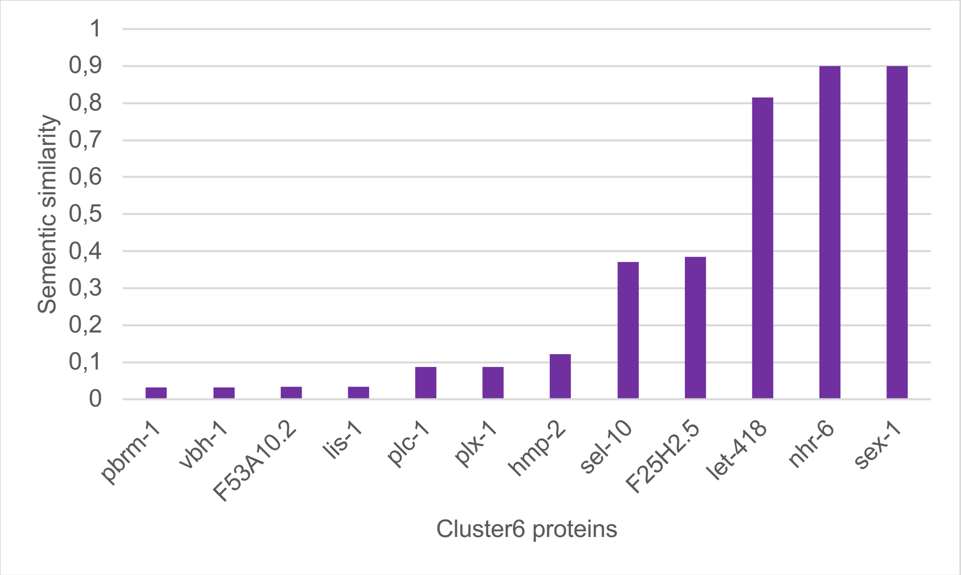

Figure S4. GO semantic similarity between cluster 6 proteins and LRGs.

Supplementary references

De Vaux, V., Pfefferli, C., Passannante, M., Belhaj, K., von Essen, A., Sprecher, S.G., Müller, F. and Wicky, C. The Caenorhabditis elegans LET-418/Mi2 plays a conserved role in lifespan regulation. *Aging Cell* **12**, 1012-1020 (2013).

Gillis, J. and Pavlidis, P. “Guilt by association” is the exception rather than the rule in gene networks. *PLoS Comp Biol* **8**, e1002444 (2012).

Zhao, C. and Wang, Z. GOGO: An improved algorithm to measure the semantic similarity between gene ontology terms. *Sci Rep*, **8**, 15107 (2018).
